# Monthly Record of Deaths in the City of Buffalo

**Published:** 1857-03

**Authors:** C. L. Dayton

**Affiliations:** Health Physician


ART. IX.—*Report of Mortality in Buffalo for the month of Jan.,* 1857. By C. L. DAYTON, M. D., Health Physician.

| DISEASES. | No. | Males. | Females. |
|---|---|---|---|
| Apoplexia, | 1 | | 1 |
| " Pulmonalis, | 1 | | 1 |
| Accidens, | 3 | 1 | 2 |
| Burned, | 1 | | 1 |
| Cholera Infantum, | 2 | | 2 |
| Croup, | 2 | 1 | 1 |
| Debilitas Senilis, | 4 | 2 | 2 |
| Diarrhœa, | 3 | 3 | |
| Dysinteria, | 2 | | 2 |
| Dissipatio, | 1 | | 1 |
| Diabetes Mellitus, | 1 | | 1 |
| Erysipelas, | 1 | 1 | |
| Enteritis, | 2 | 1 | 1 |
| Epilepsy, | 1 | 1 | |
| Eclampsia, | 15 | 10 | 5 |
| Febris Typhoid, | 1 | 1 | |
| " Typhus, | 1 | 1 | |
| " Puerperarum, | 2 | | 2 |
| " Scarlatina, | 39 | 19 | 20 |
| " " Sequel of, | 2 | 2 | |
| Fracture Cranii, | 1 | 1 | |
| Gangrena Pulmonalis, | 1 | 1 | |
| Hydrops, | 5 | 3 | 1 |
| Hyperæmia Pulmonalis, | 2 | 2 | |
| " Cerebralis, | 1 | | 1 |
| Hydrocephalus, | 8 | 6 | 2 |
| Ignotus, | 23 | 9 | 12 |
| Morbus Cordis, | 1 | | 1 |
| " Hepatis, | 1 | 1 | |
| Marasmus, | 7 | 4 | 3 |

REGISTER OF MORTALITY—Continued.

| DISEASES. | No. | Males. | Females. |
|---|---|---|---|
| Parturitio, | 1 | | 1 |
| Premature Birth, | 3 | 1 | 2 |
| Pertussis, | 2 | 1 | 1 |
| Pneumonitis, | 6 | 3 | 3 |
| Peritonitis Puerperarum, | 1 | | 1 |
| Pleuritis Chronica, | 1 | 1 | |
| Paralysis, | 1 | 1 | |
| Rupture of the Aorta, | 1 | 1 | |
| Suicide by taking opiates, | 1 | 1 | |
| Still-born, | 6 | 2 | 2 |
| Tuberculosis Pulmonalis, | 18 | 10 | 8 |
| " et Laryngitis Chronica, | 1 | 1 | |
| Total, | 177 | | |

SEXES.

| | |
|---|---|
| Males, | 92 |
| Females, | 80 |
| Sex not given, | 5 |
| Total, | 177 |

AGES.

| | | | |
|---|---|---|---|
| Still-born, | 6 | Between 10 years and 20 years, | 8 |
| 1 day, | 3 | " 20 " " 30 " | 9 |
| 1 day and 30 days, | 14 | " 30 " " 40 " | 15 |
| Between 1 month and 6 months, | 15 | " 40 " " 50 " | 7 |
| " 6 months and 12 " | 17 | " 50 " " 60 " | 12 |
| " 1 year " 3 years, | 35 | " 60 " " 70 " | 4 |
| " 3 " " 5 " | 16 | " 70 " " 80 " | 2 |
| " 5 " " 10 " | 10 | " 80 " " 90 " | 1 |
| | | " 90 " " 100 " | 0 |
| Total number under 10 years, | 116 | Total number over 10 years, | 58 |
| Ages not given, | 3 | Total, | 177 |

NATIVITIES.

| | | | |
|---|---|---|---|
| American, (white,) | 105 | Canadian, | 1 |
| " (colored,) | 2 | French, | 2 |
| German, | 33 | Scotch, | 0 |
| Irish, | 16 | Holland, | 0 |
| English, | 4 | Country not given, | 14 |
| Total, | 177 | | |

